# Relationship between Gross Motor Coordination and Health-Related Fitness in Serbian Preschool Children

**DOI:** 10.3390/children11080933

**Published:** 2024-07-31

**Authors:** Nenad Stojiljković, Nebojša Trajković, Doroteja Rančić, Mihai Olanescu, Adrian Suciu, Danut Popa

**Affiliations:** 1 Faculty of Sport and Physical Education, University of Nis, 18000 Nis, Serbia; nenad@fsfv.ni.ac.rs (N.S.); nele_trajce@yahoo.com (N.T.); doroteja.r@yahoo.com (D.R.); 2 Faculty of Automotive, Mechatronics and Mechanical Engineering, Technical University of Cluj-Napoca, 400641 Cluj-Napoca, Romania; adrian.suciu@mdm.utcluj.ro (A.S.); ilie.popa@mdm.utcluj.ro (D.P.)

**Keywords:** motor competence, childhood, physical activity, gross motor skills, correlation

## Abstract

Background: The relationship between motor coordination and physical fitness in preschool children is of significant interest due to its implications for overall health and development. This study aims to investigate the correlation between gross motor coordination, as assessed by the Körperkoordinationstest für Kinder (KTK), and health-related physical fitness components. Methods: A cross-sectional study, involving 139 preschool-aged children, was conducted. Physical fitness was assessed using the PREFIT fitness test battery, which includes measures of flexibility, muscular strength, speed/agility, and cardiorespiratory fitness. Motor coordination was evaluated using the KTK test, comprising four subtests. Results: Pearson correlation analysis revealed weak to strong positive and negative correlations between motor coordination and various physical fitness measures, including flexibility (r = 0.402; *p* = 0.01), muscular strength (r = 0.178; r = 0.487; r = 0.601; *p* < 0.05), speed/agility (r = −0.742; *p* = 0.01), and endurance (r = 0.539; *p* = 0.01). Additionally, hierarchical regression analysis demonstrated significant influence of motor coordination on physical fitness, explaining a notable percentage of the variance across different fitness components (3.2–55%). Conclusions: The findings underscore the importance of motor coordination in shaping physical fitness levels in preschool children. Promoting motor coordination skills early in childhood may have long-term benefits for overall health and fitness.

## 1. Introduction

Motor competence, encompassing motor coordination (MC) and fundamental movement skills (FMS), plays a crucial role in preschool children’s physical development and overall health [1]. Therefore, preschool-aged children are at a critical stage of developing physical and motor skills, and significant progress in their motor capabilities can be seen, including both fine and gross motor abilities [2]. Gross motor coordination represents a child’s general motor abilities, which are essential for daily activities and the development of various motor skills [3]. Moreover, children with high levels of motor competence tend to perceive physical tasks as less difficult, engage more frequently in physical activities, and, as a result, achieve higher levels of physical fitness [4]. Health-related physical fitness comprises several components, including cardiovascular endurance, muscular strength, muscular endurance, flexibility, and body composition [5]. Each of these components is independently linked to positive health outcomes such as reduced risks of cardiovascular disease, obesity, and mental health issues, and improved bone density [6,7]. 

There is a clear correlation between motor competence and physical fitness, particularly as children grow into adolescence and adulthood [8]. Adolescents with higher motor-coordination levels typically have better fitness status compared to their peers with lower motor-competence proficiency [9]. Studies have consistently demonstrated positive associations between motor coordination and various physical fitness components [9,10,11,12,13] and along with an inverse relationship with body composition [14]. This suggests that enhancing motor-coordination proficiency can lead to improved physical fitness levels, contributing to better overall health throughout life [4,9]. Recent studies have continued to highlight the significant relationship between motor competence and physical fitness in preschool children. For instance, research indicates that higher levels of motor coordination are associated with better physical fitness outcomes, including enhanced cardiovascular endurance, muscular strength, and flexibility [15]. In addition, comprehensive assessments reveal that children engaged in multiple organized physical activities exhibit superior motor skills and physical fitness compared to those participating in single-sport activities [16]. The association between motor coordination and physical fitness underscores the importance of early and diverse physical activity programs which can enhance physical fitness and reduce the risk of health issues such as obesity and cardiovascular diseases as children grow into adolescence and adulthood [15]. Although numerous studies have confirmed the existence of an association between motor coordination and physical fitness, some authors disagree with this conclusion. In the study by Kurt, Canli & Prieto-González [17], no significant association was found between motor competence and physical performance in kindergarten children. However, inconsistencies in the use of the same tests and type of scores present a problem. Some studies utilize a single measure from each test battery or prefer raw scores, while others use standardized scores. This variation can lead to discrepancies in findings, as raw scores may be confounded by age [18] whereas standardized scores for expected performance change over time [8].

These inconsistencies necessitate further investigation into how different measures from test batteries demonstrate associations. The existing literature indicates a need for more consistent and comprehensive assessments to better understand relationships between physical fitness and motor coordination. The rationale for this study lies in addressing the inconsistencies of previous research by utilizing a comprehensive and standardized assessment approach. By employing consistent testing measures, this study would provide clearer insights into the relationship between gross motor coordination and health-related physical fitness in children, thereby offering a more reliable foundation for developing targeted interventions to enhance children’s physical development. Therefore, understanding this relationship is essential, especially as it evolves with age, to develop effective strategies for enhancing health outcomes in children [18]. Given the significant role that motor coordination and physical fitness play in children’s overall development, it is crucial to address these gaps. In this regard, the aim of this study was to examine the relationship between gross motor coordination and health-related physical fitness in preschool children.

## 2. Materials and Methods

### 2.1. Study Design and Procedures

#### 2.1.1. Study Design

This study was conducted as a cross-sectional study aiming to examine the association between gross motor coordination and health-related fitness in preschool children. The study concentrated on a group of Serbian children, all under the age of 7.

#### 2.1.2. Procedures

To determine the required sample size for this study, we performed an a priori calculation using the G*Power v3.1 programme (Bonn, Germany, Bonn FRG, University of Bonn, Department of Psychology). G*Power (1 ß > 0.9; effect size = 0.3; α = 0.05) was used to determine the sample size based on two of the most important components of fitness in our study: grip strength and shuttle run. Based on the G*Power programme, the required sample size was 111 people. In total, 139 children participated in this study. Characteristics of the sample are shown in Table 1, including the mean values and the standard deviation of children’s age, body height, body mass, and body mass index. 

This study included the oldest groups of children in kindergartens (children aged 6 to 7 years old). In Europe, children typically start primary school at the age of 6, and their education spans 9 grades. However, in Serbia, children begin first grade at the age of 7 and undergo 8 grades of primary education. The respondents were selected based on specific inclusion criteria: children aged only up to 7 years old, attending kindergarten regularly, and having no diagnosed physical or cognitive disabilities that could affect their performance in physical fitness tests. Data collection for this study was conducted in a kindergarten setting. Trained researchers in physical fitness assessments carried out the measurements, ensuring proficiency in tools and techniques. The testing took place in a well-lit and spacious indoor area during the spring, with the temperature maintained at a comfortable 22 °C. The children wore their regular play clothes and participated in a 20-min warm-up session involving light exercises and games. Therefore, testing was conducted simultaneously, with all children tested in the morning between 9 a.m. and 11 a.m. over multiple days to prevent fatigue while maintaining consistent conditions. All tests were administered uniformly, ensuring reliable indoor conditions and consistent measurement across different days. To ensure that participants were adequately prepared and to minimize potential variability due to unfamiliarity with the testing procedures, we conducted a familiarization session prior to the actual testing. Parental or guardian approval was obtained for the inclusion and involvement of each child in the study. 

### 2.2. Measures

#### 2.2.1. Physical Fitness

Physical fitness was measured using the PREFIT fitness test battery [19] to determine flexibility, muscular strength, speed, and cardiorespiratory fitness. 

Flexibility was assessed with a sit-and-reach test, which specifically measures the flexibility of the lower back and hamstring muscles. The task demanded of the participant was to sit on the floor with bare feet, legs completely stretched and feet flat against the sit-and-reach box (Eveque Leisure Equipment Ltd., Unit 11 Wincham Avenue, Northwich, Cheshire, UK). Then, participants had to reach as far forward along the measuring line as they could. The farthest location and maximum result attained on the two tries, with a break of 30 s, was measured in centimeters. 

Muscular strength was assessed using three tests: standing long jump, sit-ups, and handgrip strength. The standing long jump was used to determine the explosive power of the lower limbs. The participants stood slightly apart behind the takeoff line. The hips, knees, and ankles were barely bent, and the participants jumped as far as possible with hand swings. The distance from the takeoff line to the spot where the heels touched the ground was measured to the nearest 0.1 cm. The highest score recorded over two trials was used as the measure of explosive strength. The “sit-ups in 30 s” test was used to assess abdominal and hip flexor muscles. At the sound signal, participants elevated their chest to a vertical position before returning to the floor. As a result of this test, the total number of correctly performed sit-ups within a 30 s period was recorded (Casio HS-80TW Stopwatch, Casio Europe GmbH, Norderstedt, Germany).

To assess hand muscle strength, the Takei handgrip digital dynamometer (Takei Scientific Instruments Co., Ltd., Tokyo, Japan) was used. Two trials were performed with the dynamometer, standing with the elbow flexed at 90°. Participants performed the test both with their dominant and non-dominant hand, and the better result was recorded. A rest period of 30 s was provided between individual attempts for both dominant and non-dominant hand. Also, verbal encouragement was provided throughout the grip-strength tests to support participants and maximize their performance.

Speed/agility was measured using a 4 × 10 m shuttle-run test (SRT), which was conducted in an indoor gymnasium. This test required participants to run and turn as quickly as possible between two parallel lines marked 10 m apart on the floor, covering a total distance of 40 m. To make the test more suitable for children, it was modified by having two evaluators stationed at each end. Participants had to touch the evaluator’s hand (positioned behind the line) and then return to the starting point at maximum speed. The fastest time from two attempts, with a break of two minutes, was recorded in seconds with a help of a stopwatch (Casio HS-80TW Stopwatch, Casio Europe GmbH). 

Cardiorespiratory fitness was also assessed by a shuttle-run test, adapted to assess physical fitness levels in preschool children [20]. Participants ran back and forth between two lines 20 m apart, guided by an audio signal. The test concluded when a child failed to reach the lines in sync with the signal twice consecutively or stopped due to exhaustion. To accommodate the young age of preschoolers, the initial speed was reduced to 6.5 km/h from the original 8.5 km/h, and two evaluators ran alongside a smaller group of 4–8 children to maintain the pace. An audio track with signals starting at 6.5 km/h, increasing by 0.5 km/h each minute, was recorded for the adapted test.

#### 2.2.2. Motor Coordination

To assess the motor coordination of children, the Körperkoordinationstest für Kinder (KTK) was used. KTK is a widely used assessment tool to measure gross motor coordination skills in children. Developed by Kiphard & Schilling [21], the KTK test has become a valuable instrument in assessing motor skills, identifying motor deficits, and monitoring progress in children’s motor development. 

The KTK test comprises four subtests, each designed to evaluate different aspects of gross motor coordination: The first task is to walk backward three times along three different balance beans. The maximum score of 72 is determined by counting the steps taken without falling, with a maximum of 8 steps each try. The goal is to assess a child’s ability to maintain balance and coordination while performing a motor task that challenges their proprioception and spatial awareness. The second task is jumping sideways, which assesses a child’s ability to perform lateral jumps over a wooden board as quickly and accurately as possible within a specified 15 s time frame. One-leg jumping over a foam barrier made up of smaller pieces of foam that are 5 cm high is the third subtest. The maximum score of each leg is 39 points (three points for a successful first try, two points for a successful second try, and one point for a successful third try). The fourth subtest activity involves moving from one wooden platform to another while gripping and moving the first platform sideways as much as possible for 20 s. The score was calculated by adding the results of two trials together.

Each subtest produces a raw score based on the child’s performance, which is then converted into a standardized score according to their age and gender. Normative data tables were utilized to transform the raw performance score of every test item into a standardized motor quotient (MQ), which was then adjusted for gender and age. These standardized scores enable comparison with established norms. 

### 2.3. Statistical Analysis

The data were analyzed using the Statistical Package for the Social Sciences (SPSS) (20.0, SPSS Inc., Chicago, IL, USA). Descriptive statistics were calculated for each variable, showing the mean and standard deviation The normality of the data distribution was confirmed using the Kolmogorov–Smirnov test. The Pearson correlation was used to analyze the relationship between motor coordination and motor abilities. Pearson’s correlation classifies relationships as weak (±0.10), moderate (±0.30), or strong (±0.50) [22]. Further, to determine the impact of motor coordination on the physical fitness of children, hierarchical regression analysis was applied (motor coordination in block 1 while controlling for age and BMI effects in block 2) The statistical significance was set at *p* ≤ 0.05. 

### 2.4. Ethics

The study was conducted in accordance with the Declaration of Helsinki and approved by the Ethics Committee of the Faculty of Sport and Physical Education, University of Nis (No. 04-428/2 approved 23 March 2024). 

## 3. Results

The arithmetic means and standard deviations of the physical fitness variables (flexibility–sit and reach; muscle strength–standing long jump, sit ups, and handgrip; speed/agility—4 × 10 m running; endurance–shuttle-run test), as well as the motor quotient from the Körperkoordinationstest für Kinder, are shown in Table 2.

As the data distribution was shown to be normal through the application of the Kolmogorov–Smirnov test, Pearson’s correlation was used to demonstrate the relationship between the physical fitness variables and the motor quotient from the KTK. Considering the correlation coefficient obtained from Pearson’s analysis, we can conclude that there is a weak to strong positive correlation as well as a negative correlation between the physical fitness variables and motor coordination (Table 3). Positive correlations were observed between the following variables: motor quotient of KTK and handgrip showed weak correlation (r = 0.178; *p* = 0.036); the motor quotient of KTK and the flexibility sit-and-reach test (r = 0.402; *p* = 0.01), as well as the motor quotient of KTK and sit-ups (r = 0.487; *p* = 0.01), showed moderate correlation. Strong correlation was found between the motor quotient of KTK and the endurance shuttle-run test (r = 0.539; *p* = 0.01) and between the motor quotient of KTK and the standing long jump (r = 0.601; *p* = 0.01). A weak negative correlation was shown between body mass index and motor coordination (r = −0.249; *p* = 0.01), while between motor coordination and the 4 × 10 m running test, strong negative correlation was found (r = −0.742; *p* = 0.01).

Hierarchical regression analysis (Table 4) was used with variables grouped into blocks based on similarity. The results suggest that the two models are statistically significant (*p* = 0.01) and that the introduction of the subsequent group of variables increases the percentage of explained variance: block 1, consisting of MQ KTK; controlling for height, weight, and body mass index in block 2. A hierarchical linear regression for the sit-and-reach test showed that motor coordination and block 1 were significant (R^2^ = 0.161; *p* = 0.01) and explained 16.1% (R^2^ × 100) of the variance. However, the addition of body composition measures did not significantly increase the prediction of motor coordination (R^2^ = 0.211) and the percentage of variance increased only for 5% (R^2^ × 100), making a total of 21.1% explained variance. Regarding the standing long jump, motor coordination demonstrated significant influence on physical fitness (R^2^ = 0.361; *p* = 0.01), which explained 36.1% of the variance. Furthermore, block 2 increased the explained variance by 3.4% (R^2^ = 0.034), ensuring that body-composition parameters did not significantly influence the findings of this test. Taking into account the influence of motor coordination on the other tests, we can conclude that the findings are very similar: block 1 (MQ KTK) showed statistically significant influence on the sit-ups, the handgrip, the 4 × 10 m, and the shuttle-run test (*p* < 0.05), explaining 23.7%, 3.2%, 55%, and 29.1% of the variance, respectively. Block 2 (body height, body weight, and body mass index) showed a statistically significant influence only in the handgrip strength test (R^2^ = 0.459; *p* = 0.01) while in all other tests it was not statistically significant (*p* > 0.05)

## 4. Discussion

This study aimed to analyze the correlation between motor coordination and health-related physical fitness, and additionally to ascertain whether and how much motor coordination influences physical fitness in childhood. The main findings suggested that motor coordination showed weak to strong correlation with all physical fitness measures including flexibility, strength, speed/agility, and endurance. Regarding the influence of motor coordination on the physical fitness of children, it can be seen that motor coordination significantly affects the physical fitness of children of that age, explaining a high percentage of the variance, while the parameters of body composition do not significantly contribute to the percentage of explained variance.

The correlation analysis revealed that motor coordination, as assessed by the KTK test, exhibited weak to strong positive correlations with different physical fitness measures. Specifically, handgrip strength demonstrated a weak positive correlation (r = 0.178; *p* = 0.036), while flexibility, assessed through the sit-and-reach test, and sit-ups performance showed moderate correlations (r = 0.402; *p* = 0.01 and r = 0.487; *p* = 0.01, respectively). Notably, the strongest positive correlations were found between motor coordination and both endurance, measured by the shuttle-run test (r = 0.539; *p* = 0.01), and explosive strength, measured by the standing long jump (r = 0.601; *p* = 0.01). In accordance with these results are results of a similar study conducted by Batez et al. [23], in which the long jump also demonstrated the strongest correlation with motor coordination (r = 0.480). The results obtained in this way can be justified by the fact that explosive strength, particularly as measured by the standing long jump, requires a high degree of neuromuscular coordination [24]. In addition, the standing long jump has been recognized as an excellent proxy for assessing lower-body muscular fitness and motor coordination due to its requirement for coordinated lower-body forces in a horizontal direction [25]. Therefore, findings from these studies are consistent with previous research that emphasizes the importance of various physical fitness components in the development of motor skills during childhood [4,26]. Moreover, the obtained results suggest that enhancing motor coordination can directly impact a child’s overall physical fitness, which in turn can promote healthier and more active lifestyles. In contrast to these results, the findings of the study conducted by Kurt, Canli & Prieto Gonzalez [17] found no significant relationship between the participants’ physical performances and motor coordination (*p* > 0.05). This could be explained by the possibility that the fitness assessment tests used might have been too complex for children of that age. Regarding parameters of body composition, a weak negative correlation was observed between body mass index (BMI) and motor coordination, indicating that higher BMI might be associated with poorer motor coordination (r = −0.249; *p* = 0.01). This aligns with the understanding that excessive body weight can hinder motor performance due to biomechanical constraints and reduced physical activity levels [27]. Additionally, a higher BMI can lead to a greater body mass that needs to be supported and moved during physical activities [28]. This added weight can hinder efficient movement patterns and reduce agility and speed, crucial components of motor coordination [28]. Children with higher BMIs may struggle with tasks requiring balance, quick directional changes, and coordinated muscle actions due to the increased mechanical load on their bodies [29]. Also, this reinforces the need to focus on motor skill development rather than just body composition when addressing physical fitness in early childhood. Contrary to the results of this study are the findings of Catenassi et al. [22], which indicate that the performance of children in tasks which involve gross motor skills did not significantly correlate with body composition parameters (*p* > 0.05). 

The hierarchical regression analysis further elucidated the significant influence of motor coordination on physical fitness. Block 1, incorporating MQ KTK, explained a different level of variance in physical fitness (from 3.5% to 55%). This highlights the critical role motor competence plays in enhancing children’s physical fitness. A few studies have been conducted that align closely with our findings. The results obtained from these studies corroborate with our conclusions, further validating the significant influence of motor coordination on physical fitness in children. For instance, research by Behan et al. [1] utilized hierarchical regression analysis to demonstrate the contribution of motor coordination on fitness components. They reported that motor coordination explained from 11.3% to 50.2% of the variance in physical fitness, which is consistent with the significant impact of motor coordination found in our study. A study by Robinson et al. [9] found that motor-competence measures accounted for 55% of the variance in physical fitness in children and adolescents (R² = 0.55). These studies collectively reinforce our findings, showing a consistent trend of physical fitness significantly impacting motor coordination, with some variation in the extent of the explained variance. 

However, the addition of body composition measures (block 2) did not significantly increase the explained variance. This suggests that while physical fitness significantly influences motor coordination, body composition parameters such as BMI have a relatively minor role in this context. This finding contrasts with some previous research where body composition was considered a significant factor. For example, Malina, Bouchard, and Bar-Or [27] discussed the importance of body composition in children’s physical fitness and motor coordination. Higher BMI can negatively affect motor coordination because excess weight can hinder movement efficiency and make physical tasks more challenging. This can lead to lower participation in physical activities and consequently poorer physical fitness. Yet, our study suggests that while body composition may play a role, it is not as critical as the direct measures of motor coordination. These findings underscore the importance of promoting motor coordination in early childhood to physical fitness. Improved motor coordination can lead to higher levels of physical activity participation, creating a positive feedback loop that fosters overall physical health and fitness [4]. Additionally, the study supports the notion that early interventions targeting physical fitness and motor competence can have long-lasting benefits, potentially mitigating the risk of developing chronic diseases later in life [15].

Despite the valuable insights gained from this study, several limitations need to be acknowledged. Firstly, the cross-sectional design of the study restricts our ability to infer causality between physical fitness and motor coordination. While we can establish associations, we cannot determine whether improvements in physical fitness led to enhanced motor coordination or vice versa. Longitudinal studies are necessary to establish causal relationships and to understand the long-term effects of motor coordination on physical fitness. Secondly, the sample size of 139 children, while sufficient for initial analysis, may not be large enough to generalize the findings to a broader population. Additionally, the study was conducted within a specific age (children up to seven years old), and the results may not be applicable to younger or older children. Future studies should include a larger and more diverse sample to enhance the generalizability of the findings. Furthermore, the study did not account for potential confounding variables such as socioeconomic status, nutritional status, and levels of physical activity outside of the structured assessments. These factors could influence both physical fitness and motor coordination and should be considered in future research to control their effects.

## 5. Conclusions

In conclusion, this study contributes to the growing body of evidence that motor coordination plays a crucial role in the development of physical fitness in children. The study examines the relationship between gross motor coordination and health-related physical fitness in Serbian preschool children. Using the Körperkoordinationstest für Kinder (KTK) to assess motor coordination and the PREFIT fitness test battery for physical fitness, the study found significant correlations between motor coordination and various fitness components, including flexibility, strength, speed/agility, and endurance. The results highlight that motor coordination substantially influences physical fitness, explaining a considerable portion of the variance in fitness levels. Promoting motor coordination skills early in childhood may thus yield long-term benefits for overall health and fitness. This study reinforces the importance of incorporating various fitness activities into early childhood programs to support physical fitness development. Future research should aim to explore longitudinal effects to better understand how early motor-coordination interventions can influence physical fitness and overall health outcomes throughout the lifespan.

## Figures and Tables

**Table 1:** Sample characteristics.

*n* = 139	Mean	SD	K-S
AGE	6.58	0.94	0.134
BH (cm)	126.81	5.91	0.957
BM (kg)	26.33	5.27	0.061
BMI (kg/m^2^)	16.26	2.25	0.077

BH—body height; BM—body mass; BMI—body mass index; K-S—Kolmogorov–Smirnov test.

**Table 2:** Arithmetic mean and standard deviation for all tested variables.

*n* = 139	Mean	SD	K-S
SR (cm)	34.65	4.40	0.164
SLJ (cm)	123.93	18.78	0.247
SU (freq.)	18.01	4.80	0.364
HG (kg)	13.19	2.44	0.254
4 × 10 m (s)	14.62	1.41	0.404
SHR	18.83	8.61	0.128
MQ KTK	0.27	19.09	0.785

SR—sit and reach; SLJ—standing long jump; SU—sit ups; HG—handgrip; 4 × 10—4 × 10 speed test; SHR—shuttle run; MQ KTK—motor quotient from the Körperkoordinationstest für Kinder; K-S—Kolmogorov–Smirnov test.

**Table 3:** Pearson correlation for body mass index, physical fitness, and motor coordination.

	BMI	SR	SLJ	SU	HG	4 × 10 m	SHR
SR	0.081						
SLJ	−0.277 **	0.332 **					
SU	−0.093	0.249 **	0.407 **				
HG	0.418 **	0.000	0.185 *	0.138			
4 × 10 m	0.194 *	−0.300 **	−0.677 **	−0.515 **	−0.307 **		
SHR	−0.362 **	0.033	0.506 **	0.361 **	0.034	−0.551 **	
MQ KTK	−0.249 **	0.402 **	0.601 **	0.487 **	0.178*	−0.742 **	0.539 **

*—correlation significant at 0.05 level; **—correlation significant at 0.01 level; SR—sit and reach; SLJ—standing long jump; SU—sit-ups; HG—handgrip; 4 × 10—4 × 10 m speed test; SHR—shuttle run; MQ KTK—motor quotient from the Körperkoordinationstest für Kinder.

**Table 4:** Hierarchical regression analysis.

	F	ΔR^2^	R^2^	*p*
SR				
Block 1	26.338	0.155	0.161	0.01
Block 2	8.958	0.187	0.211	0.01
SLJ				
Block 1	77.464	0.357	0.361	0.01
Block 2	21.865	0.377	0.395	0.01
SU				
Block 1	42.626	0.232	0.237	0.01
Block 2	11.379	0.231	0.254	0.01
HG				
Block 1	4.469	0.025	0.032	0.03
Block 2	32.41	0.476	0.491	0.01
4 × 10				
Block 1	167.746	0.547	0.550	0.01
Block 2	46.841	0.574	0.583	0.01
SHR				
Block 1	56.191	0.286	0.291	0.01
Block 2	18.092	0.331	0.351	0.01

F—F statistic; R^2^—coefficient of determination (square); ΔR^2^—adjusted R^2^; *p*—statistical significance; SR—sit and reach; SLJ—standing long jump; SU—sit-ups; HG—handgrip; 4 × 10—4 × 10 m speed test; SHR—shuttle run; MQ KTK—motor quotient from the Körperkoordinationstest für Kinder; Block 1—MQ KTK; Block 2—body height, body weight, and body mass index.

## Data Availability

Data are contained within the article.
